# Improved Pathologic Diagnosis—Forecasting the Future in Glioblastoma

**DOI:** 10.3389/fneur.2017.00707

**Published:** 2017-12-19

**Authors:** David J. Pisapia, Rajiv Magge, Rohan Ramakrishna

**Affiliations:** ^1^Department of Pathology and Laboratory Medicine, Weill Cornell Medical College, New York, NY, United States; ^2^Department of Neurology, Weill Cornell Medical College, New York, NY, United States; ^3^Department of Neurological Surgery, Weill Cornell Medical College, New York, NY, United States

**Keywords:** glioblastoma, GBM, molecular diagnostics, IDH mutation, MGMT promotor, whole exome sequencing

## Introduction

The 2007 WHO classification of central nervous system (CNS) tumors was nearly devoid of incorporating molecular parameters as official diagnostic criteria. One of the few exceptions was the incorporation into the definition of atypical teratoid rhabodoid tumors the associated inactivation of *INI1/nSNF5* in “virtually all cases” (1). In 2014, a consensus conference of neuropathologists held in Haarlem, Netherlands, determined that an update to the WHO was necessary in order to incorporate many of the newly discovered molecular underpinnings of CNS tumors, even prior to the release of a bona fide new edition of the WHO (2). Moreover, this conference outlined a new approach to CNS tumor diagnostics that included both histologic and molecular findings, ultimately yielding an “integrated diagnosis.” The culmination of the initial phase of this paradigm shift is the updated 2016 WHO classification of CNS tumors (3).

It is important to note that even in this updated 2016 WHO classification, there is continued reliance on the cardinal histologic features of microvascular proliferation and necrosis as the gold standard diagnostic criteria to distinguish GBM from lower-grade infiltrating astrocytomas. However, the update for the first time incorporates molecular features into the GBM diagnostic rubric at the highest levels. *IDH* mutational status has become part of the diagnosis itself, with entire sections devoted to *Glioblastoma, IDH-mutant* versus *Glioblastoma, IDH-wild type*. Moreover, high-grade infiltrating astrocytomas characterized by mutation of *H3F3A* K27M are now denoted explicitly as *Diffuse midline glioma, H3K27M-mutant*.

Even for entities that do not currently have molecular parameters as required for defining criteria, the WHO now places commonly associated alterations prominently within the initial paragraph description of each entity, perhaps as a prelude to future editions of the WHO, whereby, these molecular alterations will indeed also become required criteria. For example, particularly relevant to GBM, the variant “epithelioid glioblastoma” is noted to be characterized frequently by BRAF V600E mutations, a factor, which has generated interest regarding its relationship to pleomorphic xanthoastrocytoma. Similarly, giant cell glioblastoma is noted to differ from classical *de novo* GBM in its propensity to harbor *TP53* mutations, *AURKB* alterations, and infrequent amplification of *EGFR* (3, 4).

One cannot overstate the importance of incorporating somatic molecular alterations as gold standard diagnostic criteria. This represents an increasingly prominent paradigm shift within the field of pathology and one that has profound implications for the entire workflow of patient management. It is important to understand how these new molecularly defined diagnostic categories came about, how they impact the concept of histological grading, and how they portend similar changes in the future.

## The Emergence of Molecular Diagnostics in GBM

The last decade has been the witness to significant advances in our molecular understanding of GBM. As the pilot project for The Cancer Genome Atlas (TCGA) Research Network, a large-scale sequencing effort of adult GBM uncovered recurrent patterns across tumors while, at the same time, revealing remarkable heterogeneity within the disease. The project established core signaling pathways implicated as drivers in GBM, including the receptor tyrosine kinase (RTK), p53, and retinoblastoma pathways (5). Datasets generated by the TCGA, which have been continually expanded upon to date, have permitted the emergence of molecular subclassifications of GBM based on unsupervised clustering algorithms using both genetic and epigenetic parameters.

In work by Verhaak et al. (6), subtypes of GBM including *Proneural, Neural, Mesenchymal, and Classical* were proposed and correlated with recurrent, often mutually exclusive alterations. For example, those tumors that harbored *EGFR* amplification clustered within the *Classical* group, those with *IDH* mutations or *PDGFRA* amplification clustered within the *Proneural* group, and those with *NF1* alterations were enriched within the *Mesenchymal* group. Indeed, the *Proneural* group was found to have two subclasses: those tumors tightly associated with *IDH* mutations and the glioma-CpG island methylator phenotype (G-CIMP) that is mutually exclusive with those harboring *PDGFRA* alterations (6).

Later work incorporating methylation data from a broader demographic, including pediatric patients led to the clustering of GBM into six categories including *IDH, RTK-I (PDGFRA), RTK-II (Classic), Mesenchymal, K27*, and *G34*. While the *RTK-II and Mesenchymal* groups were enriched for tumors within the *Classical and Mesenchymal* grouping from the Verhaak classification, the newer methylation data allowed for further subclassification of tumors that would have fallen within Verhaak’s *Proneural* group (7). In particular, aided by the inclusion of pediatric gliomas enriched in H3 mutations, the methylation data yielded four additional GBM subclassifications: those with *IDH* mutations (also previously described as G-CIMP tumors), *PDGFRA* amplifications, and those with alterations in the *H3F3A/HIST1H3B/C* genes (either K27 or G34 mutations). Finally, methylation data has further contributed to potential molecular subclassification by identifying a subset of tumors with GBM-defining histological criteria that tend to cluster together molecularly with pilocytic astrocytoma (a low-grade, non-infiltrative astrocytoma) (8). These tumors frequently demonstrate alterations in *BRAF, NF1*, and *NTRAK1/2* alterations rather than those associated with other classes of GBM (8). Among the molecular features characteristic of these proposed subclassifications, the presence or absence of *IDH* mutations in GBM has been one of the earliest molecular changes to become incorporated explicitly into any diagnostic designation in the WHO. It is, therefore, instructive to explore this development in greater detail.

## IDH Mutation

While *IDH* mutation is most characteristic of lower-grade infiltrating gliomas, including astrocytomas and oligodendrogliomas, it was in fact the study of GBM that initially led to the discovery of this recurrently altered gene (9). Since it was noted that GBMs with this alteration typically arose from lower-grade tumors, these findings quickly led to the discovery that *IDH* mutations are found in the vast majority of lower-grade infiltrating gliomas (10–14).

Indeed, correlating IDH-status with clinical and demographic parameters of GBM patients clearly indicated the existence of two distinct clinicopathologic entities: (1) those tumors that tend to arise in younger patients as lower-grade astrocytomas and progress over time to GBM-IDH-mutant versus (2) those that tend to arise in older patients and more rapidly lead to death, namely GBM*-*IDH-wild type. Thus, these two entities overlap with our historic understanding of “secondary” and “*de novo*” GBM, respectively. Reciprocally, anaplastic astrocytomas (AA), defined simply as proliferative infiltrating astrocytomas that do not exhibit microvascular proliferation or necrosis in histologically examined material, also display variable outcomes with many behaving as poorly as GBM. Specifically, AA’s that are wild type for IDH are identical to GBM from a survival standpoint. Therefore, IDH status has become a valuable prognosticator independent of histologically defined grades in the WHO (13).

A second major consequence of practical and diagnostic importance related to IDH mutational status is clarification regarding high-grade oligodendroglial tumors and GBM. In particular, previous conceptions of mixed glial tumors exhibiting both astrocytic and oligodendroglial components no longer have a welcome place in the updated WHO, assuming that appropriate molecular tests are available and have been performed. In fact, all infiltrating gliomas should now be regarded as belonging to three broad, unambiguous, molecularly defined groups based only on combined IDH status and 1p/19q co-deletional status (Table 1).

**Table 1:** Combined IDH and 1p/19q co-deletional status (Table 1).

	IDH mutant	IDH wild type
1p/19q codeleted	Oligodendroglioma	^a^
1p/19q non-codeleted	Astrocytoma, IDH-mutant	Astrocytoma, IDH-wild type

*^a^Tumors demonstrating an unbalanced translocation involving chromosomes 1 and 19 (resulting in a relative deletion of 1p and 19q) *in the absence of IDH mutation* are extremely rare to virtually nonexistent (14)*.

This paradigm is further supported by the lack of tumors demonstrating biclonality with respect to these two parameters (13). The result is that several entities have been dropped entirely from the updated WHO, or at least their use has been strongly discouraged. These include *oligoastrocytoma, anaplastic oligoastrocytoma*, and the so-called *GBM with oligodendroglial component*. These entities represented a heterogeneous group of tumors with poor interneuropathologist concordance that are better defined based on the IDH-1p/19q schema (13, 15).

## TERT Promoter

Interestingly, additional mutations can be superimposed upon the IDH-1p/19q rubric completely independent of histology to yield further clinically distinguishable categories. For example, evidence shows that classification of all infiltrating gliomas based on IDH, 1p/19q, and the mutational status of the *TERT* promoter yields clinically relevant subclasses (14). The *TERT* promoter is mutated very frequently in oligodendrogliomas and IDH-wild type GBM, and is only infrequently mutated in *IDH-mutated, non-codeleted* tumors (i.e., IDH-mutant astrocytomas) where it is seen in only 4% of cases. A classification system based on these three parameters yields five categories (accounting for >97% of infiltrating gliomas) with *TERT* status permitting further refinement of prognostics (Table 2). For example, among IDH-mutated tumors that demonstrate grade IV histologic criteria, survival is worse in the presence of a *TERT* promoter mutation (14). Investigations utilizing *TERT* promoter mutational status in conjunction with additional molecular parameters, for example, MGMT promoter methylation status, are underway in an effort to generate additional prognostically relevant patient subgroups (16). Recent data have demonstrated that the survival advantage associated with MGMT promoter methylation may also require *TERT* promoter mutation (17).

**Table 2:** Classification system.

	IDH mutant	IDH wild type
1p/19q codeleted + TERT intact	Rare (~2.6% of gliomas)	Virtually non-existent
	Most with oligodendroglial phenotype	

1p/19q non-codeleted + TERT intact	Astrocytoma (mostly lower grade)	Triple negative
		Astrocytoma (mostly GBM)

1p/19q codeleted + TERT mutated	Triple positive	Virtually non-existent
	Oligodendroglioma	

1p/19q non-codeleted + TERT mutated	Astrocytoma (mostly lower grade)	Astrocytoma (mostly GBM)
	Worse prognosis among *GBM, IDH-mutant*	

*Based on data from Ref. (14)*.

## Next-Generation Sequencing Using a Targeted Approach

Increasingly over the last 5–10 years, molecular assessment has become integrated into the workup of GBM. Tests that have become commonplace and are now regarded as standard of care include sequencing to detect *IDH1* or *IDH2* mutations (further discussed below). A second test is the assessment of the *MGMT* promoter for the presence of methylated CpG sites. While there are several methodologies to accomplish this, one method involves PCR-based assays following pretreatment with bisulfite, which specifically alters unmethylated base pairs over those that are methylated and allows methylation status to be detected in the amplified product. The methylation status of this promoter in turn correlates with tumor response to the alkylating agent temozolomide.

With the advent of relatively cheap methodologies for targeted next-generation sequencing, many laboratories now employ sequencing as part of the routine workup for glioblastoma in order to assess genetic loci in addition to the IDH genes. In some pathology departments, a panel of genes is used to target known cancer hotspots. These panels are typically designed to interrogate genes that are broadly affected across multiple cancer types, but they also may be more tailored for CNS disease (18). At our institution, the current panel designed for solid tumors utilizes the Ion Torrent AmpliSeq™ Cancer Hot Spot Panel that covers known hot spots over 50 genes. Genes on the panel that are of potential relevance to GBM include *IDH1, IDH2, BRAF, EGFR, PDGFRA, PIK3CA, PTEN*, and *TP53*. In some circumstances, for example, the panel is employed if there is a suspicion for a possible *BRAF* mutation based on the presence of epithelioid morphological characteristics. Larger panels on the order of hundreds of genes are also increasingly becoming available to pathology departments. At our institution, we are currently implementing and validating the Oncomine^®^ panel. Originally developed at the University of Michigan, the Oncomine^®^ panel sequences hotspots over 73 genes as well as copy number variants over an additional 75 genes and RNA-based fusion detection involving 22 genes (19). Other institutions, such as Memorial Sloan Kettering Cancer Center, have developed their own panels such as *MSK-IMPACT*, a custom designed hybridization capture-based panel interrogating 410 genes (as of 2015) (20). Commercially available tests provided by private companies, such as the FoundationOne^®^ test, present additional opportunities to sequence tumor tissue for those centers without in-house sequencing capability or even as a supplement for those centers that do. FoundationOne^®^ analyzes the coding region of 315 genes and introns from 28 genes (21).

In contrast to whole exome sequencing (WES), more targeted panels have the advantage of permitting a greater depth of coverage, or the number of times any particular base is sequenced and aligned to the reference genome, a factor which is particularly important when the amount of starting material may be small. Infiltrating gliomas involving the brainstem or thalamus, for example, are often only assessed *via* stereotactic biopsy, and tissue yield may be very limited. For such specimens, it is of crucial diagnostic importance to distinguish between those tumors with IDH mutation, those wild type for IDH, and those with H3 K27M mutations, all of which now correspond to explicitly recognized distinct diagnostic entities in the updated WHO. While immunohistochemistry may detect IDH mutations and K27M mutations (specific antibodies are available for both, requiring only a single section of tissue), sequencing would ideally be used to confirm the immunohistochemical findings, especially in the absence of positive staining.

## Wes and Other Methodologies

In addition to targeted sequencing panels, broader approaches to assess the cancer genome can also be employed. For example, the goal of the Englander Institute for Precision Medicine (IPM) at Weill Cornell Medicine is to examine all coding regions of the tumor genome relative to germline (i.e., WES). The idea is to detect both alterations that are commonly and recurrently altered across patients for a given tumor type, but also to detect uncommon somatic mutations and copy number alterations within an individual patient’s tumor that might be used to guide personalized, targeted therapy (22). The program also characterizes the RNA profile of tumor cells in order to detect, for example, high expression levels of targetable cancer drivers or potential fusion partners. The clinically relevant advantages and disadvantages of designing a program that is based upon more targeted approaches versus WES, or even broader approaches such as whole genome sequencing, are still being studied and are not yet fully understood. Indeed, there are often arguments that can be made in favor of each technology and the winning solution may likely be some combination of each approach. For example, the *TERT* promoter has been described as a recurrently altered locus in GBM with diagnostic and clinical implications for patients. It would not be covered in a pure WES paradigm that only interrogates coding regions, so, the addition of a targeted approach for this particular locus may be necessary. Moreover, even within the coding regions of the genome, depending on the design of the WES assay, certain coding regions may routinely suffer from poor coverage, and a targeted approach for those regions could be used to supplement the overall WES assay. As a final point, relatively broad interrogation of the tumor genome, for example, through larger targeted panels or WES, can reveal insight into the overall mutational burden of a tumor that itself may have clinically relevant implications. For example, tumor cells with defective mismatch repair mechanisms and a high mutational burden may express a greater number of neoantigens with the potential to elicit an immunological response. Such information, in addition to direct immunohistochemical assessment of immune checkpoint-related proteins, may increasingly be used in the context of GBM to identify tumors with potentially increased susceptibility to immune checkpoint inhibitors such as nivolumab (23–25).

## Methylation Profiling

It is becoming increasingly recognized that many CNS tumors can be successfully characterized and/or classified by examining the epigenetic profile of a tumor, instead of the DNA base pair sequence itself. For example, in CNS tumors, various tumor classification schemas have been proposed based either solely on methylation profiling or with methylation profiling as one of several parameters. Ependymomas have recently been characterized by methylation profiling as have CNS embryonal tumors (24, 26). In GBM, methylation patterns alone can generate robust clusters, some of which correlate with particular subclasses based on mutational spectra such as those with IDH mutation, H3 alterations, or receptor kinase alterations (7, 27). As discussed above, the methylation status of specific loci can also demarcate tumors into clinically relevant classes, such as those bearing MGMT promoter methylation, which can predict response to alkylating agents and may be used to guide therapeutic decision-making.

## Conclusion

We are at a fascinating point in GBM diagnostics. *The pace at which our ability to accrue high-resolution molecular information about individual tumors has far outpaced our ability to act on this information in a clinically relevant way*. We lie at crossroads wherein classification of GBM will need to be iteratively revisited as the classes themselves begin to impact therapeutic decision-making and ultimately patient outcomes. We will need to assess the best methodologies that offer the greatest clinical yield to the patient. In the context of next-generation sequencing, the issues of both depth and breadth of coverage are highly dependent on the employed techniques. It remains to be seen which technology or combination thereof can offer the highest-yield, clinically relevant results for the greatest number of patients in a way that can be implemented successfully into our health-care system infrastructure. It will also be important to analyze the extent to which differing platforms such as targeted approaches, WES, RNA-based, and epigenetic profiling, offer redundant information with respect to clinical outcome. Such an analysis can inform best practice usage of potentially limited biopsy tissue.

While histology still very much remains ingrained in the diagnostic workflow as a gold standard from which further molecular testing and clinical decisions follow, as a field we should consider freeing ourselves from this paradigm and consider whether resources could better be spent entirely in the molecular realm. For example, does detection of an H3-K27M mutation offer sufficient information to subclassify an H3-mutated tumor and/or to determine the best course of action? If not, how often does further histological molecular investigation result in clinically actionable or clinically meaningful results? Will MR spectroscopic techniques, tumor detection in peripheral blood or CSF, and other techniques obviate the need for more invasive approaches to tumor? The answers to these questions themselves, of course, are not static. They will change iteratively as information impacts treatment, the design of clinical trials, and the application of drugs, many of which are yet to be designed or FDA approved.

An argument for the implementation of precision medicine programs is that while any particular molecular alteration may be found in only one or a few patients, for that patient, the discovery could be life changing. However, at the programmatic level, a more appropriate metric might be one that assesses whether the design of the precision medicine pipeline yields results that are beneficial at the population level and with reasonable cost. Much more time will be required before we can start to assess the efficacy of high-resolution molecular approaches in GBM diagnostics and clinical care. Moreover, this assessment will require further iterative interaction between state of the art molecular assay resulting, clinical trial design, therapeutic application, and clinical outcomes.

For now, what should a patient in the United States with glioblastoma expect? How should patients, surgeons, pathologists, and oncologists proceed with the potentially limited tissue that is obtained? These questions are quite complicated and dependent upon practical issues that may vary dramatically on a case-by-case basis. Even so, there are several principles that can be considered.

First, it is crucial to remember that the primary objective of analyzing resected tissue is to inform the subsequent management. While standard medical practice currently dictates that we prioritize “rendering a diagnosis” following guidelines like those published by the WHO, this presumes that current diagnostic schemas are perfect in their intended ability to prognosticate, stratify, and inform. The reality is necessarily more different and dependent on updating diagnostic criteria to reflect the most current basic science and clinical data. An important example is the lingering issue of the relationship between grade and IDH status in infiltrating astrocytomas. Should oncologists and patients breathe a sigh of relief when a proliferative and infiltrating IDH-wild type astrocytoma does not exhibit microvascular proliferation in a biopsy sample? No. The point is, assuming we are confident that we are dealing with an infiltrating astrocytoma, IDH status may be the only diagnostically relevant piece of information needed. If this information could reliably be obtained through non-invasive means such as MR-spectroscopy in the future, so be it.

At a minimum then, IDH status must be assessed for all infiltrating gliomas. In most cases, this requires a relatively inexpensive immunohistochemical test on a single section of tissue. For those patients younger than 55 years, sequencing should be performed when *IDH1* (R132H) immunostaining is negative in order to detect less frequent but functionally equivalent mutations in *IDH1* (e.g., R132G) or *IDH2* (i.e., R172H). Beyond this, in cases that are IDH-mutant and for which oligodendroglioma is within the differential diagnosis, 1p/19q assessment is mandatory. While some practitioners may feel obligated to obtain 1p/19q deletional status for all cases of infiltrating glioma, the probability of discovering this alteration in tumors that are clearly astrocytic morphologically (particularly for those demonstrating immunohistochemical evidence of TP53 and ATRX mutations) is quite low.

Other tests that have become standard of care, but are not diagnostic *per se*, include assessment of MGMT promoter methylation status. This test is performed for all infiltrating gliomas as a way to predict response to temozolomide.

Additional testing, for example, the assessment of EGFRvIII status, should arguably first be prioritized based on requirements of clinical trials. Many trials require repeat testing of tissue in a centralized laboratory as part of eligibility determination. While in theory, this may reduce biases introduced by laboratory testing variability across sites, limited tissue may be subjected to redundant testing if advance tissue triage planning is not carefully coordinated by oncologists, patients, and pathologists. The problem is exacerbated by the understandably frequent desire of patients with GBM to seek opinions at multiple institutions, many of which have an institution-centric rather than patient-centric approach to patient data management. Testing redundancy due to the partially overlapping information garnered by analyses offered by academic centers and commercial entities presents a complex set of problems ripe for optimization by health information experts, pathologists, and others.

Further molecular profiling in the form of targeted next-generation sequencing, WES, and whole genome sequencing is an additional consideration, may identify individualized cancer drivers that could guide targeted treatment after standard therapies have failed. However, by the time standard therapies have failed, the tumor has been subjected to and likely changed by those standard therapies as well as by time and the intrinsic clonal evolution of the tumor. Thus, the original molecular profile obtained by a primary resection may have reduced relevance to the rational selection of treatment options at recurrence. At some institutions, further biopsy sampling may be performed at recurrence with the sole purpose of obtaining up-to-date molecular information even when surgical cytoreductive therapy is not indicated.

Indeed, there is sufficient heterogeneity across GBM to justify further molecular interrogation as a way to more comprehensively characterize the disease in an individual patient. Whether this information will impact patient outcomes at the population level, at reasonable cost, is a fundamental question that remains unanswered at this time. Rigorous interinstitutional data collection, annotation of patient data, and more time for analysis are all required.

Finally, if we revisit the idea that the goal of tissue analysis is guide clinical management, is it possible to consider bypassing the traditional concept of “diagnosis” itself? High throughput tumor-derived *in vitro* or xenograft drug screening modalities could theoretically identify combinations of agents that are most effective in treating a disease without explicitly defining the disease in the first place. There are many limitations to these approaches, including designing tumor models representative of the endogenous tumor environment. Moreover, the approach presumes at least some preliminary characterization (i.e., a rudimentary diagnosis of sorts) to determine that further treatment is even warranted.

Many laboratories are engaged in the design of protocols to examine tumor tissue in this way, including with the use of two- and three-dimensional culture systems, patient-derived xenografts in rodents, and human stem cell-derived or iPSC-derived cerebral organoids implanted with patient-derived tumor cells.

In conclusion, we are at a remarkable inflection point in the practice of oncology—the sweet spot between information and patient management—hidden within a complex multidimensional space where big data, technology, patient heterogeneity, tumor heterogeneity, and financial considerations collide. Managing the acquisition and utilization of this information *via* a reasonable and universally applied algorithm is a central challenge that will depend upon the continued cooperation and dedication of all tasked with this enormous problem.

## Author Contributions

RR is the corresponding author. DP and RM have contributed equally to this paper.

## Conflict of Interest Statement

The authors declare that the research was conducted in the absence of any commercial or financial relationships that could be construed as a potential conflict of interest.
